# Author Correction: High-order harmonic generation in an organic molecular crystal

**DOI:** 10.1038/s41467-026-69230-5

**Published:** 2026-02-04

**Authors:** Falk-Erik Wiechmann, Samuel Schöpa, Lina Bielke, Svenja Rindelhardt, Serguei Patchkovskii, Felipe Morales, Maria Richter, Dieter Bauer, Franziska Fennel

**Affiliations:** 1 Institute for Physics, University of Rostock, Rostock, Germany; 2 Department Life, Light & Matter, University of Rostock, Rostock, Germany; 3 Max Born Institute, Berlin, Germany

**Keywords:** High-harmonic generation, Electronic properties and materials

Correction to: *Nature Communications* 10.1038/s41467-025-65975-7, published online 10 November 2025

In the version of the article initially published, two references were missing and have now been added as refs. 41 and 42: “Jacquemin, D. et al. Extensive TD-DFT Benchmark: Singlet-Excited States of Organic Molecules. *J. Chem. Theory Comput*. **5**, 2420–2435 (2019)” and “Ang, S. T., Pal, A. & Manzhos, S. Comparison of optical absorption spectra of organic molecules and aggregates computed from real frequency dependent polarizability to TD-DFT and the dipole approximation. *J. Chem. Phys*. **149**, 44114 (2018)”. Additionally, original refs. 47, 48 have been renumbered as refs. 39, 40. These corrections have been made to the HTML and PDF versions of the article.

