# Supplementary material for: High-order harmonic generation in an organic molecular crystal
Source: Nat Commun. 2025 Nov 10;16:9890. doi: 10.1038/s41467-025-65975-7 (PMC12603074; doi:10.1038/s41467-025-65975-7)
Supplement: Supplementary file 1 — Supplementary Information [file 41467_2025_65975_MOESM1_ESM.pdf]

Supplementary Information

High-Order Harmonic Generation in an Organic Molecular Crystal

Falk-Erik Wiechmann[1,2], Samuel Schöpa[1], Lina Bielke[1], Svenja Rindelhardt[1,2], Serguei Patchkovskii[3], Felipe Morales[3], Maria Richter[3], **Dieter Bauer[1,2], Franziska Fennel[1,2](corresponding authors: Franziska.fennel@uni-rostock.de and Dieter.bauer@uni-rostock.de)**

[1]Institute of Physics, University of Rostock, 18051 Rostock, Germany.

[2]Department Life, Light & Matter, University of Rostock, 18051 Rostock, Germany.

[3]Max Born Institute, Max Born Str. 2a, 12489 Berlin, Germany.

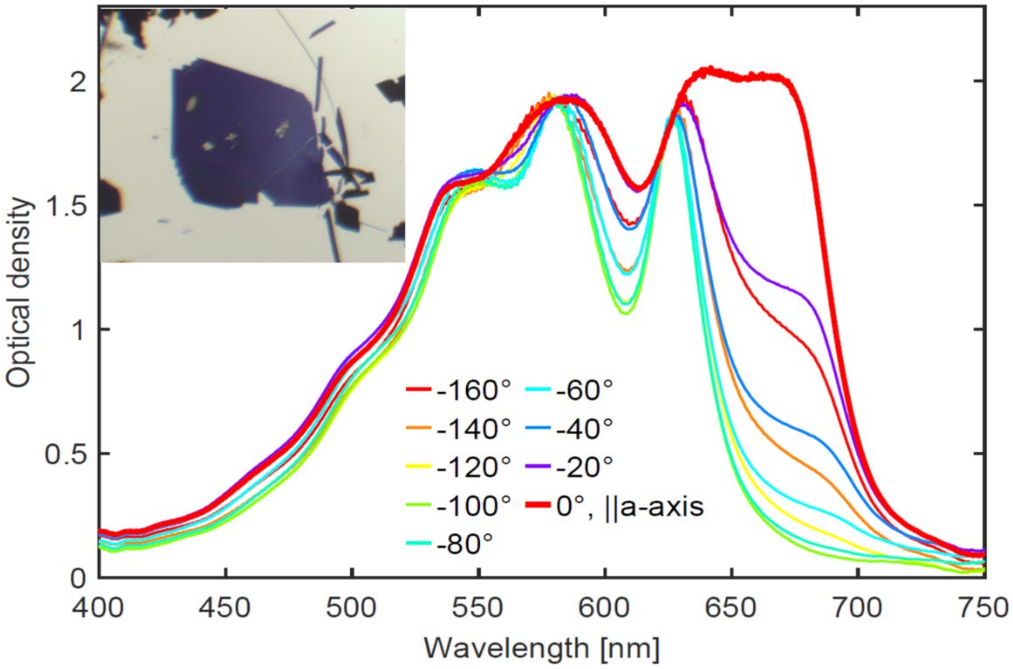

Supplementary Figure 1: Absorption spectra for different white light polarization directions relative to the crystal axes. The red spectrum exhibits maximum absorption at 670 nm, which is the transition in direction of the **a**-axis. The inset shows a microscope picture of the crystal.

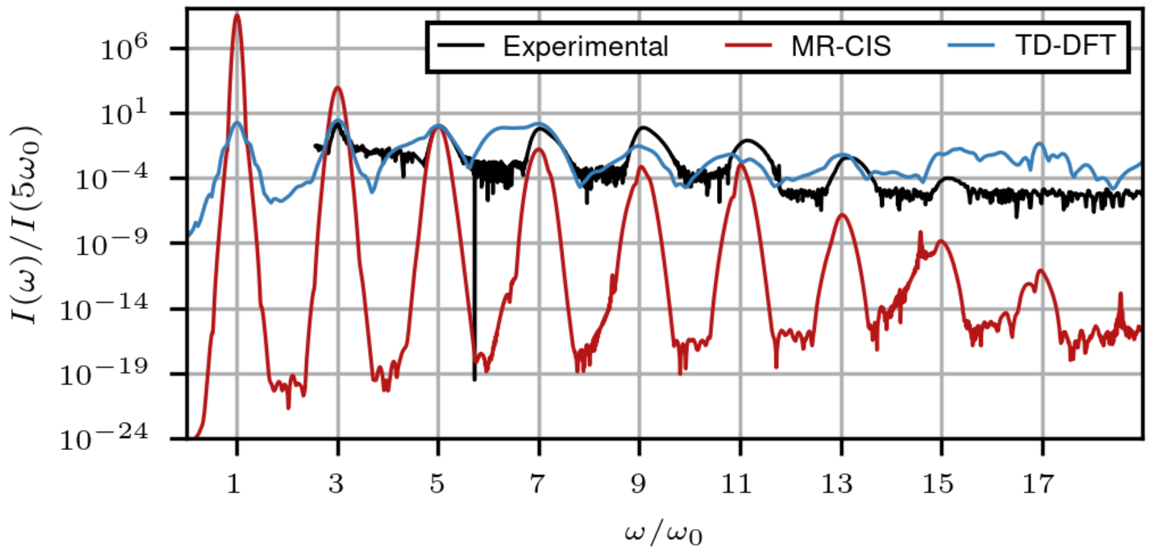

Supplementary Figure 2: Comparison of the experimentally measured harmonics (black) with calculated harmonics obtained for the case of pairs of non-interacting pentacene molecules based on the MR-CIS single molecule calculations (red) and for the full periodic pentacene crystal based on the TD-DFT calculations (blue). All spectra are normalized to the fifth order as the orders 5 to 15 were measured with the same spectrometer. The harmonic spectra correspond to the laser polarization of 130° for all three cases displayed. Note that a direct comparison between the calculated harmonics requires caution as they are based on two fundamentally different systems with different approximations: gamma-point-only crystal TD-DFT calculations on a real-space grid with a local XC functional and single molecule quantum chemistry calculations using a localized basis. However, the comparison reveals a distinct difference between the relative scaling between the low and high orders. When comparing the drop of yield between low and high orders, the experimental and the TD-DFT crystal spectra show good agreement. In the spectrum for the pairs of non-interacting pentacene molecules, the drop between order three and order 13 is more significant, about 10 orders of magnitude. Such a strong drop is clearly not observed in experiment irrespective of a potential wavelength sensitive detection efficiency in the experiment.

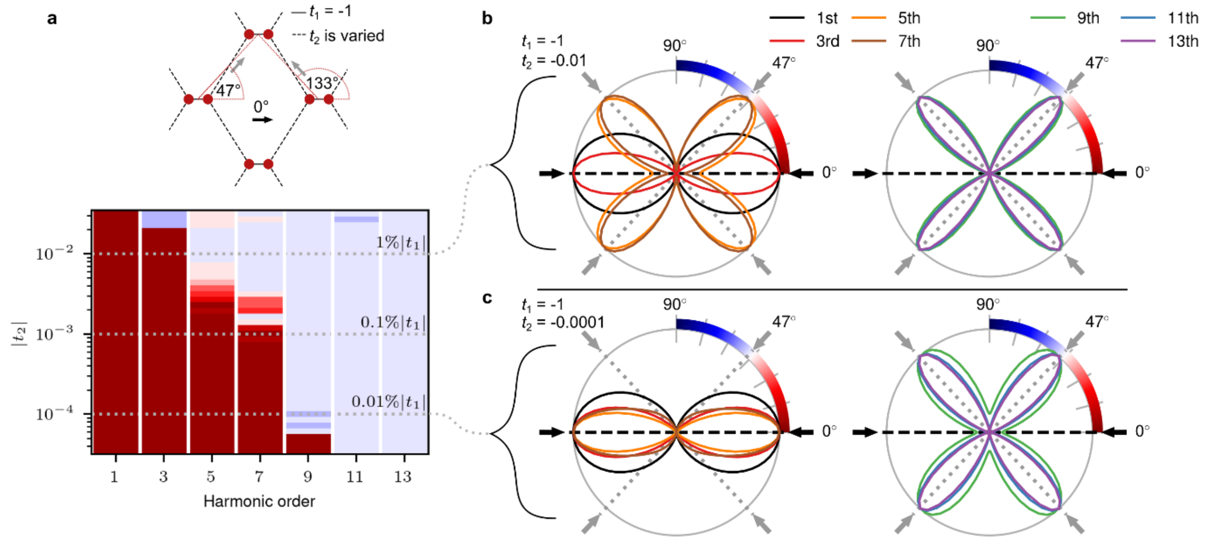

Supplementary Figure 3: Polarization dependence of the harmonics obtained with the tight-binding model in Fig. 4c as a function of the intermolecular coupling parameter t_2. **a**, Crystal structure of the tight-binding model from Fig. 4c alongside the 2D color plot from Fig. 4d showing the laser polarization φ that maximizes the harmonic yield for different harmonic orders as a function of the intermolecular coupling t_2**.** The colors in the 2D color plot are extracted from the polar plots in **b, c** on the right as the angles at which the t_2-dependent harmonic yields maximize, as indicated by the surrounding colorbars in **b** and **c. b, c,** Normalized yields of harmonics 1 to 13 for two selected values of t_2, t_2=1%|t1| (**b**) and t_2=0.01%|t1| (**c**), as a function of laser polarization φ. The colorbar at the polar plots maps the polarization angle, for which the individual yields are maximized, to the color that is shown in **a.**
